# Living with an imperfect cell wall: compensation of *femAB *inactivation in *Staphylococcus aureus*

**DOI:** 10.1186/1471-2164-8-307

**Published:** 2007-09-04

**Authors:** Judith Hübscher, Andrea Jansen, Oliver Kotte, Juliane Schäfer, Paul A Majcherczyk, Llinos G Harris, Gabriele Bierbaum, Matthias Heinemann, Brigitte Berger-Bächi

**Affiliations:** 1 Institute of Medical Microbiology, University of Zürich, Zürich, Switzerland; 2 Institute for Medical Microbiology, Immunology and Parasitology, University of Bonn, Bonn, Germany; 3 Institute of Molecular Systems Biology, ETH Zürich, Zürich, Switzerland; 4 Seminar for Statistics, ETH Zürich, Zürich, Switzerland; 5 Division of Infectious Diseases, Department of Internal Medicine, Centre Hospitalier Universitaire Vaudois, Lausanne, Switzerland; 6 AO Research Institute, Davos, Switzerland; 7 School of Medicine, University of Wales Swansea, Swansea, UK

## Abstract

**Background:**

Synthesis of the *Staphylococcus aureus *peptidoglycan pentaglycine interpeptide bridge is catalyzed by the nonribosomal peptidyl transferases FemX, FemA and FemB. Inactivation of the *femAB *operon reduces the interpeptide to a monoglycine, leading to a poorly crosslinked peptidoglycan. *femAB *mutants show a reduced growth rate and are hypersusceptible to virtually all antibiotics, including methicillin, making FemAB a potential target to restore β-lactam susceptibility in methicillin-resistant *S. aureus *(MRSA). *Cis*-complementation with wild type *femAB *only restores synthesis of the pentaglycine interpeptide and methicillin resistance, but the growth rate remains low. This study characterizes the adaptations that ensured survival of the cells after *femAB *inactivation.

**Results:**

In addition to slow growth, the *cis*-complemented *femAB *mutant showed temperature sensitivity and a higher methicillin resistance than the wild type. Transcriptional profiling paired with reporter metabolite analysis revealed multiple changes in the global transcriptome. A number of transporters for sugars, glycerol, and glycine betaine, some of which could serve as osmoprotectants, were upregulated. Striking differences were found in the transcription of several genes involved in nitrogen metabolism and the arginine-deiminase pathway, an alternative for ATP production. In addition, microarray data indicated enhanced expression of virulence factors that correlated with premature expression of the global regulators *sae*, *sarA*, and *agr*.

**Conclusion:**

Survival under conditions preventing normal cell wall formation triggered complex adaptations that incurred a fitness cost, showing the remarkable flexibility of *S. aureus *to circumvent cell wall damage. Potential FemAB inhibitors would have to be used in combination with other antibiotics to prevent selection of resistant survivors.

## Background

The peptidoglycan structure of *Staphylococcus aureus *is a dynamic, three-dimensional meshwork consisting of multiple layers of glycan strands that are crosslinked through peptide bridges. It determines the bacterial shape and confers protection against the high internal turgor. Characteristic for the staphylococcal peptidoglycan is the long and flexible pentaglycine interpeptide, which branches off the ε-amino group of the L-lysine of the peptidoglycan stem peptide. The pentaglycine interpeptide is synthesized in a sequential fashion by the FemABX family of nonribosomal peptidyl transferases, which use glycyl-tRNA as a glycine donor. While FemX (synonym: FmhB) adds the first glycine, FemA and FemB add Gly_2,3 _and Gly_4,5_, respectively [1-4]. Although structurally and functionally related, these factors cannot substitute for one another [5]. Growth of mutants with a shortened interpeptide is strongly impaired [2]. They display a massive reduction in cell wall crosslinking, aberrant septum formation, and hypersusceptibility to antibiotics including all β-lactams [1,2]. In methicillin-resistant *S. aureus *(MRSA), methicillin resistance is completely abolished upon inactivation of *femA*, suggesting that the monoglycine peptidoglycan is a very poor substrate for the native penicillin-binding proteins (PBPs) as well as for the low affinity PBP2a encoded by *mecA*, which confers resistance to β-lactams. FemX and/or FemA were therefore regarded as potential targets for novel antibacterial agents, which could restore β-lactam susceptibility in MRSA [6]. While FemX was shown to be essential [7], *femAB *null mutants were postulated to require a secondary, yet uncharacterized compensatory or suppressor mutation(s) chr* to stabilize the cell [6]. The phenotype of a *femAB *null mutant thus reflects not only the consequences of the inactivation of the *femAB *operon, but additionally the effects due to the postulated compensatory mutation(s). These compensatory events or adaptations are of potential interest, as they may tell us about the interrelationship between cell wall synthesis and other cellular mechanisms. By re-introducing the *femAB *wild type allele in *cis*, the compensatory effects were separated from those due to the *femAB *inactivation. This allowed us to study the consequences of the adaptation events in the presence of a restored pentaglycine interpeptide synthesis machinery.

## Results and discussion

### Phenotypic characterization of the *femAB*+ backcross

The *femAB *null mutant AS145 derived from the MRSA BB270 produces only a monoglycine peptidoglycan interpeptide and shows a poorly crosslinked peptidoglycan, aberrant septum formation, methicillin hypersusceptibility, and a reduced growth rate [2]. Back-transduction of the wild type *femAB *allele in *cis *by selecting for the upstream, co-transducible, silent insertion Ω2000*chr::*Tn*551*, yielding the backcross strain BB1305, restored methicillin resistance, but did not increase the growth rate [6]. Therefore, survival of AS145 was suggested to require a postulated compensatory mutation termed chr*, which was retained in BB1305. The MRSA strain BB903, which was obtained by transduction of Ω2000*chr::*Tn*551 *into BB270, represents a wild type control strain isogenic to BB1305 except for the postulated chr* mutation (Table 1).

**Table 1:** *S. aureus *strains used in this study

**Strain**	**Relevant genotype and phenotype**^a^	**Specific growth rate^b ^[1/h]**		**Source or reference**
			
		**37°C**	**42°C**	
BB270	NCTC8325 background, SCC*mec *type I; Mc-r, lysostaphin-s	nd	nd	[72]
BB903	BB270, Ω2000*chr*::Tn*551*; Mc-r, Em-r, lysostaphin-s	1.38	1.36	This study
AS145	BB270, *femAB*::*tetK*, chr*; Mc-s, lysostaphin-r	0.94	0.78	[2]
BB1305	AS145, Ω2000*chr*::Tn*551 *(*femAB*+), chr*; Mc-r, Em-r, lysostaphin-s	1.09	0.85	[6]

^a^Mc, methicillin; Em, erythromycin; r, resistant; s, susceptible.

^b^Growth in LB broth. nd, not determined.

**Figure 1:**
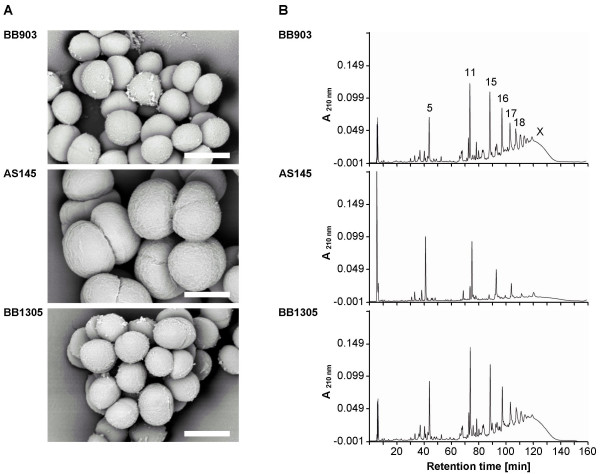
**(A) **Scanning electron microscopy pictures of cells adhering to Thermanox. The cells of the *femAB *null mutant AS145 are highly enlarged, while the *femAB+ *backcross BB1305 and the wild type BB903 show cells of the same size and appearance. The white bar corresponds to 1 μm. **(B) **Muropeptide pattern. The corresponding cell walls were digested with muramidase and subjected to reversed-phase HPLC. Major muropeptide components are numbered according to de Jonge [56]. Strains BB903 and BB1305 show a muropeptide profile characteristic of wild type *S. aureus *strains, with the highest peak in the dimeric fraction (peaks 11 and 12) and a high degree of crosslinking (peaks 15 and higher), while AS145 has the highest peak in the monomeric fraction (peaks 1 to 5) and a reduced amount of oligomeric muropeptides [2].

**Figure 2:**
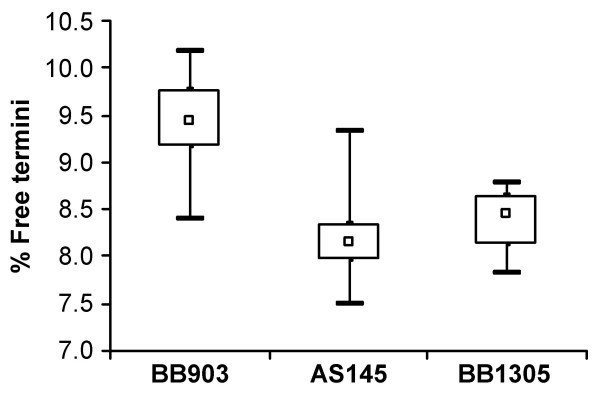
Glycan chain length. The box and whiskers diagram illustrates variations in the percentage of free reducing termini in the peptidoglycan, which are a measure of the glycan chain length. Lower values such as obtained with the *femAB *null mutant AS145 and its *femAB*+ derivative BB1305 indicate an elongated glycan chain. The square marks the median of six measurements, the lower and upper border of the box is given by the first and the third quartile, respectively. The minimum and maximum values obtained are shown by horizontal bars.

Compared to the highly enlarged cells of AS145, cells of strain BB1305, which were again able to produce a pentaglycine interpeptide, regained the same size as those of the wild type strain BB903 (Figure 1A), suggesting a regular cell separation. The muropeptide pattern of AS145 showed a highly increased amount of uncrosslinked monomeric muropeptides at the cost of the oligomeric peaks as described earlier [2]. The wild type muropeptide profile was then re-established in BB1305 as the characteristic peaks of the dimeric, trimeric, and oligomeric muropeptide fractions were indistinguishable from those of BB903 (Figure 1B). However, calculation of the percentage of free reducing termini in the peptidoglycan revealed on average slightly longer glycan chains in AS145 and BB1305 than in BB903 (Figure 2) as confirmed by two-sided *t-*test, suggesting that AS145 may have compensated for the poorly crosslinked cell wall by generating longer sugar chains.

**Figure 3:**
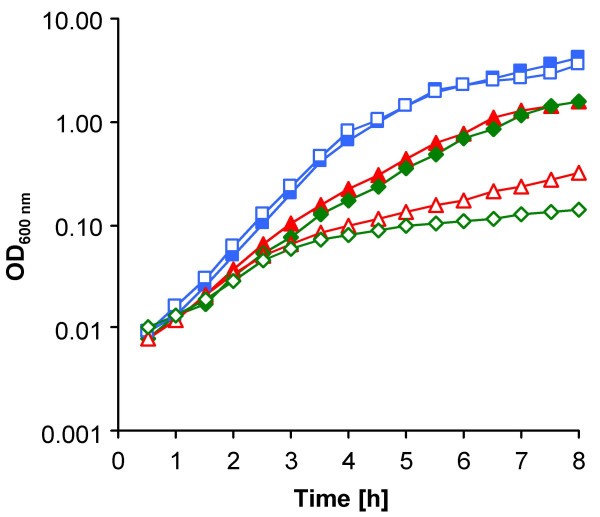
Temperature dependence of growth. Growth curves of the *femAB *null mutant AS145 (green diamonds), the *femAB*+ backcross BB1305 (red triangles), and the wild type BB903 (blue squares). The specific growth rates are indicated in Table 1. Closed symbols, growth at 37°C; open symbols, growth at 42°C.

In addition to the reduced growth rate, which may be a further strategy to cope with the cell wall defects caused by the *femAB *deletion, we found that AS145 and BB1305 also shared temperature sensitivity (Figure 3). The autolytic banding patterns and spontaneous and Triton X-100-induced autolysis, at both 37 and 42°C, were virtually identical in the slowly growing *femAB+ *backcross BB1305 and the wild type BB903 (data not shown), suggesting that there was no correlation between the autolytic behaviour of BB1305 and the reduced growth rates observed. Although the overall autolysis did not differ, subtle modulation in autolytic activities may count for the slightly increased glycan chain length.

**Figure 4:**
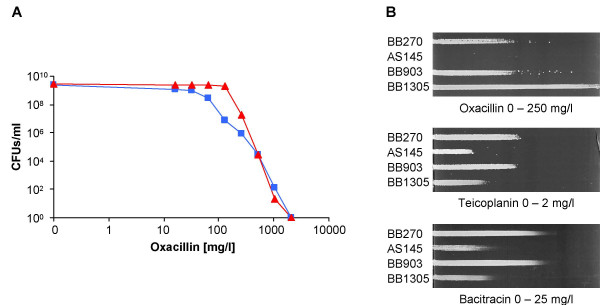
Changes in antibiotic resistance. **(A) **Population analysis resistance profiles of the *femAB*+ backcross BB1305 (red triangles) compared to the corresponding wild type MRSA BB903 (blue squares). **(B) **Antibiotic gradient plates visualizing differences in resistance levels between the *femAB *null mutant AS145, the *femAB*+ backcross BB1305, and the two MRSA strains BB270 and BB903.

The strains used in this study all carry a functional *mecA *gene and thus are MRSA. A characteristic feature of MRSA strains is the heterogeneous expression of resistance to methicillin and other penicillinase-stable β-lactams such as oxacillin, whereby the majority of cells have only a low resistance level. Upon exposure to inhibitory concentrations of β-lactams, a subpopulation with high resistance is selected. Once formed, high resistance is maintained in absence of selective pressure resulting in MRSA with homogeneous oxacillin resistance. Interestingly, detailed analyses showed that the oxacillin resistance of the *femAB+ *backcross BB1305 had become higher than that of the parental strain BB270 and of BB903, as shown in the population analysis profiles (Figure 4A) and by growth on an oxacillin gradient plate (Figure 4B). Oxacillin resistance was thus overcompensated in BB1305 and resembled homogeneous resistance as if selected by passage on inhibitory concentrations of oxacillin. In contrast, BB1305 remained hypersusceptible to teicoplanin and bacitracin (Figure 4B). The increased susceptibility to teicoplanin, which interestingly did not extend to vancomycin (data not shown), may indicate changes in the cell membrane with which the lipophilic anchor of teicoplanin interacts [8]. It is therefore likely that the bacitracin and teicoplanin hypersusceptibility of BB1305 and AS145 point to changes in the envelope and membrane organisation.

### Transcriptome analysis

Given the multiple phenotypic traits that were not complemented by restoring the pentaglycine interpeptide, changes in the genome transcription profile were expected. In a snapshot of the transcriptomes of exponentially growing cells by microarray analysis, 56 genes were reported to be downregulated in the *femAB*+ backcross strain BB1305 compared to the wild type strain BB903, and 81 genes were reported to be upregulated [see Additional file 1]. The list of differentially expressed genes was determined using the moderated *t*-statistics [9], followed by the family-wise error rate (FWER)-based *p-*value adjustment according to Holm [10], in order to guarantee high confidence in the selected genes.

**Figure 5:**
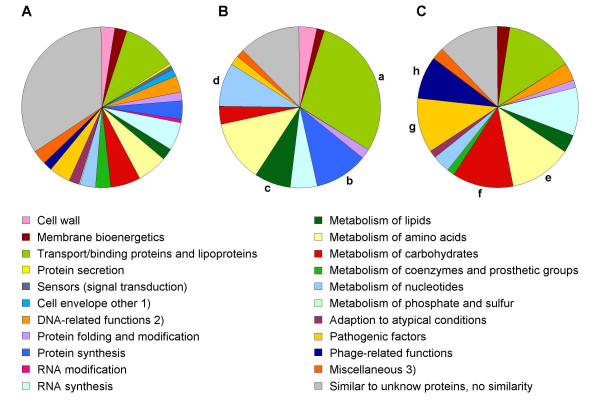
Pie charts of genes clustered according to their cellular functions. **(A) **All genes present on the microarray. **(B) **Genes detected to be downregulated in the *femAB*+ backcross BB1305. Overrepresented categories as determined by Fisher's exact test comprised transport/binding proteins and lipoproteins (a), protein synthesis (b), metabolism of lipids (c), and metabolism of nucleotides (d). **(C) **Upregulated genes in BB1305 with statistically overrepresented categories included metabolism of amino acids (e), metabolism of carbohydrates (f), pathogenic factors (g), and phage-related functions (h). 1) Cell envelope other comprises cell division, germination, sporulation, and transformation/competence; 2) DNA-related functions comprise DNA modification, repair, recombination, replication, packaging, and segregation; 3) Miscellaneous comprises also detoxification, transposon and IS.

The distribution of functional classes within the down- and the upregulated genes (Table 2) is depicted in pie charts for comparison with their occurrence within the total of genes represented on the chip (Figure 5). Among the downregulated genes in the mutant, categories significantly overrepresented as determined by Fisher's exact test comprised transport/binding proteins and lipoproteins, protein synthesis, metabolism of lipids, nucleotides and nucleic acids. Taking into account the decreased growth rate of BB1305, this finding may in part reflect the differences in growth between the two strains tested, particularly with regard to protein synthesis. In contrast, the categories that were overrepresented among the upregulated genes, i.e. metabolism of amino acids and carbohydrates, pathogenic factors, and phage-related functions, may point to changes in metabolism selected by an overall stress response to the original *femAB *deletion.

**Table 2:** Functional classification and numbers of genes that were found differentially expressed in the *femAB*+ backcross BB1305 compared to the corresponding wild type BB903 as determined by microarray analysis

**Function**^a^	**Number of ORFs**			**Overrepresentation^b^**	
	
	**Chip**	**Down**	**Up**		***p *-value**
Cell wall	63	2	0		
Membrane bioenergetics (electron transport chain and ATP synthase)	58	1	2		
**Transport/binding proteins and lipoproteins**	**254**	**16**	**11**	**down**	**< 0.001**
Protein secretion	12	0	0		
Sensors (signal transduction)	19	0	0		
Cell division, germination, sporulation, and transformation/competence	34	0	0		
DNA modification, repair, recombination, replication, packaging, and segregation	80	0	3		
Protein folding and modification	35	1	1		
**Protein synthesis**	**85**	**6**	**0**	**down**	**0.015**
RNA modification	20	0	0		
RNA synthesis	132	3	8		
**Metabolism of lipids**	**50**	**4**	**3**	**down**	**0.030**
**Metabolism of amino acids and related molecules**	**143**	**7**	**10**	**up**	**0.029**
**Metabolism of carbohydrates and related molecules**	**134**	**2**	**10**	**up**	**0.023**
Metabolism of coenzymes and prosthetic groups	70	0	1		
**Metabolism of nucleotides and nucleic acids**	**74**	**5**	**3**	**down**	**0.030**
Metabolism of phosphate and sulfur	8	0	0		
Adaptation to atypical conditions	44	0	1		
**Pathogenic factors (toxins and colonization factors)**	**97**	**1**	**9**	**up**	**0.006**
**Phage-related functions**	**43**	**0**	**7**	**up**	**0.001**
Detoxification, transposon and IS, miscellaneous	66	1	2		
Similar to unknown proteins, no similarity	811	7	10		

**Total ORFs**	**2332**	**56**	**81**		

^a^The cellular main roles are in accordance with the annotation on the DOGAN website [73].

^b^Overrepresented functions either among the down- or the upregulated genes are printed in bold and the respective *p-*values as determined by Fisher's exact test are given.

#### Metabolic differences

**Figure 6:**
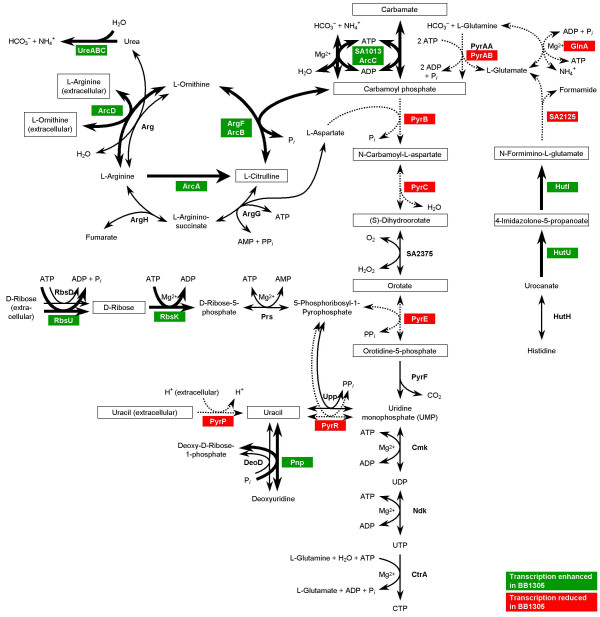
Metabolic pathways influenced by transcriptional changes observed in the *femAB+ *backcross BB1305. The corresponding gene ID numbers refer to the genome of *S. aureus *strain N315. Reactions driven by enzymes showing enhanced gene expression in BB1305 are marked with thick arrows, whereas those with reduced expression are indicated by dotted arrows. Reporter metabolites as ascertained by the method of Patil and Nielsen [67] are framed.

To uncover metabolic differences between the two strains, we made use of recent systems biology advances: In contrast to an otherwise isolated analysis of single genes, we computationally linked the transcriptional data with a recently developed metabolic network model for *S. aureus *[11] (see Methods). This procedure allowed us to consider the transcriptional differences between the two strains in a metabolic context. The mapping of transcriptional data onto a metabolic network, which underlies the employed computational algorithm, allows to identify spots (so-called reporter metabolites) around which significant regulation occurs, and thus assists in carving out metabolism-related insight from the microarray data. The top scoring reporter metabolites with *p*-values smaller than 0.05 are listed in Table 3 and an overview of pathways in which many of these reporter metabolites occur is given in Figure 6.

**Table 3:** Top scoring reporter metabolites (*p- *values < 0.05)

**Metabolism**^a^	**Metabolite**	**Number of neighbours**	**Z-score**	***p*-value**
A, E, N	Carbamoyl phosphate	5	4.341	< 0.001
N	N-Carbamoyl-L-aspartate	2	2.878	0.002
A, E, N	Carbamate	2	2.772	0.003
Co, N	Uracil	5	2.626	0.004
A	L-Citrulline	3	2.484	0.006
A	4-Imidazolone-5-propanoate	2	2.480	0.007
Co, N	Uracil (extracellular)	1	2.210	0.014
N	Orotidine-5-phosphate	2	2.182	0.015
C	Melibiose (extracellular)	1	2.092	0.018
C	Raffinose (extracellular)	1	2.092	0.018
A	N-Formimino-L-glutamate	2	2.087	0.018
L	Trihexadecanoylglycerol	1	2.075	0.019
A	L-Arginine (extracellular)	1	2.075	0.019
A	L-Ornithine (extracellular)	1	2.075	0.019
L	Phosphatidylethanolamine	2	2.047	0.020
L	Phosphatidylcholine	2	2.047	0.020
L	Choline phosphate	2	2.047	0.020
L	Ethanolamine phosphate	2	2.047	0.020
A	3-Methyl-2-oxopentanoate	3	1.980	0.024
Co	Nicotinate	2	1.979	0.024
C	Melibiose	3	1.958	0.025
A, C, L	Glycerol (extracellular)	1	1.949	0.026
N	(S)-Dihydroorotate	2	1.937	0.026
C, L	1,2-Dihexadecanoyl-sn-glycerol	7	1.924	0.027
C	N-Acetylneuraminate	1	1.907	0.028
C	Itaconate	1	1.884	0.030
C	Itaconyl-CoA	1	1.884	0.030
C	Raffinose	2	1.870	0.031
A, C, N	D-Ribose	2	1.860	0.031
A, C, L	Glycerol	4	1.804	0.036
C, Co, N	Deoxyribose	1	1.768	0.038
A	2-Aminoacrylate	1	1.746	0.040
A, N	GDP	6	1.659	0.049
N	Orotate	2	1.659	0.049
L	(R)-5-Diphosphomevalonate	2	1.650	0.049

^a^A, amino acid metabolism; C, carbohydrate metabolism; Co, metabolism of cofactors and vitamins; E, energy metabolism; L, lipid metabolism; N, nucleotide metabolism

The arginine-deiminase pathway was found to be upregulated in BB1305. This pathway imports extracellular arginine driven by the simultaneous excretion of ornithine via the arginine-ornithine antiporter ArcD (SA2426). Arginine is then converted by the arginine-deiminase (ArcA) to citrulline, which is further metabolized by the ornithine transcarbamoylase (ArcB) to ornithine and carbamoyl phosphate. The carbamate kinase (ArcC) finally breaks down the latter into ammonia and carbon dioxide yielding one ATP. This pathway is perceived to act as an ATP source under anaerobic conditions and in small colony variants (SCV) lacking a functional respiratory chain [12,13], and it is also thought to be an important player in pH homeostasis as it was also found to be expressed in biofilms [14-16]. Furthermore, depletion of arginine by the arginine-deiminase pathway, which inhibits nitric oxide production in the host, and thus both the innate and the adaptive immune responses against microbial infections, may increase staphylococcal virulence [17].

Another observation was a reduction in *glnA *expression in the *femAB*+ backcross BB1305, as was confirmed by Northern blots of the *glnRA *operon (data not shown). The glutamine synthetase (GS) GlnA produces L-glutamine from L-glutamate and ammonia using one ATP to drive the reaction. Glutamine plays a central role in nitrogen metabolism and functions as an amino group donor in many biosynthetic pathways, leading to the synthesis of histidine, tryptophan, carbamoyl phosphate, glucosamine-6-phosphate, purines, and pyrimidines. A mutation in *glnR*, which has a polar effect on *glnA*, results in a decreased GS activity and in a reduction of the amidation of the iD-glutamate of the peptidoglycan stem peptide thereby reducing methicillin resistance [18]. *glnA *downregulation in BB1305 may reflect an overall reduced requirement for nitrogen due to slowed down growth.

**Figure 7:**
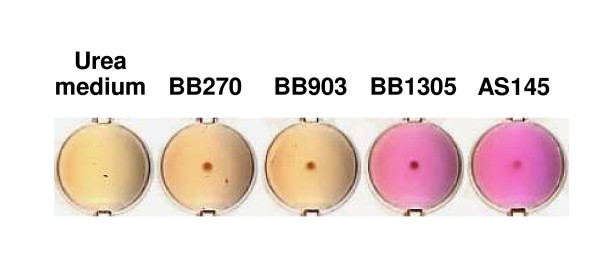
Urease production in urea-containing medium. The increase in pH resulting from the cleavage of urea is indicated by a purple colour.

Interestingly, another link to nitrogen metabolism was found in the increased amounts of *ureB *(urease beta subunit) and *ureF *(urease accessory protein UreF) transcripts displayed by strain BB1305, which was consistent with the derepression of urease production observed in AS145 and BB1305 in urea-containing medium (Figure 7). On the one hand the urease reaction supplies nitrogen and on the other hand it serves the maintenance of the pH value by formation of ammonium. Urease expression is induced during nitrogen-limited growth [19,20], and upregulation is observed in *glnA *mutants [20], growth in biofilms [14,15], and under heat shock conditions [21].

A downregulation of numerous members of the pyrimidine operon comprising *pyrAB*, *pyrB*, *pyrC*, and *pyrE *as well as of the regulator *pyrR *was detected in BB1305. The products of the *pyr *operon are involved in the *de novo *synthesis of pyrimidine nucleotides from bicarbonate and from intermediates of the central carbon metabolism or via salvage of preformed pyrimidine bases and nucleotides present in the medium. Transcription of the *pyr *operon was verified by dot blot analysis because of the large transcript expected, using a *pyrP*-specific probe (data not shown). A downregulation of the *pyr *operon was confirmed in early logarithmic growth phase at an optical density at 600 nm (OD_600_) of 0.4, however, at an OD_600 _of 1, *pyr *mRNA levels in BB1305 were the same or even higher than in BB903 (data not shown). In *Bacillus subtilis*, PyrR controls the expression of the *pyr *operon by binding to specific sequences of the *pyr *mRNA thereby leading to attenuation of transcription [22,23] in response to exogenous uracil and to intracellular UMP/phosphoribosyl pyrophosphate pools [23,24]. Richardson et al. noted that the *pyr *operon is repressed in response to nitrosative stress in *S. aureus *[25]. The upregulation of the arginine-deiminase pathway and the urease reaction as well as the downregulation of the glutamine synthesis may point to a potential action of nitrogen regulators on the *pyr *operon. Control of *pyrR *may be exerted by GlnR and TnrA [26-28], since a GlnR/TnrA consensus sequence [27,29-31] with two mismatches was identified 56 to 38 bp upstream of the coding region of *pyrR *in the public staphylococcal genomes. While according to the microarray data a reduced transcription of *pyrR *was expected, signals obtained in Northern blots using a *pyrR*-specific probe were stronger in BB1305 than in BB903 (data not shown). These were the only discrepancies between microarray data and Northern blots found in the open reading frames tested, leaving the regulation of the pyrimidine operon and the link to the regulation of nitrogen-related functions open.

#### Cell wall

Although the *femAB+ *backcross strain BB1305 produced on average slightly longer glycan chains, the transcription of *pbpB*, coding for the bifunctional PBP2 with transglycosylase activity [32], was not detected to be upregulated, as could have been expected, but downregulated. Furthermore, expression of the soluble glycosyltransferase genes, *sgtA *and *sgtB *[33,34] and of genes coding for glucosaminidases, which may contribute to an increased glycan chain length, could not be detected to be altered. These findings do not rule out a posttranscriptional control of autolytic activities by proteases [35,36], since three genes encoding proteases (i.e. the cysteine protease SspB, the zinc metalloproteinase aureolysin Aur, and the serine protease-like SplB) were significantly upregulated. In addition, the decreased expression of *dltA, dltB*, and *dltD *in BB1305 could also contribute to a lower autolytic activity due to a reduced D-alanine esterification of the teichoic acids [37].

#### Membrane and transporters

One of the major components of the membrane, lysylphosphatidylglycerol, is a product of the lysylphosphatidylglycerol synthase FmtC (synonym: MprF), which adds a positively charged lysyl residue to phosphatidylglycerol [38,39]. The *fmtC*/*mprF *gene, which belongs to the so-called *fem *and *aux *genes, and the inactivation of which reduces methicillin and bacitracin resistance [40,41], was found to be downregulated in BB1305. Reduced *fmtC *transcription may on the one hand mirror the reduced growth rate and could on the other hand, by its influence on the membrane charge, also be one of the causes for bacitracin and teicoplanin hypersusceptibility.

One fifth of the genes found differentially regulated in the *femAB*+ backcross BB1305 code for membrane-associated proteins, mainly transporters and permeases, with the majority showing a reduced transcription level. Interestingly, *opuCC*, which encodes the substrate-binding protein of a glycine betaine/carnitine/choline ABC transporter, was upregulated as could be confirmed by Northern hybridization showing a stronger transcription of the whole *opuC *operon in BB1305 than in BB903 (data not shown). The uptake of compatible solutes such as glycine betaine, choline and proline is important in osmotic stress response [42,43], and the upregulation of *opuC *may indicate an attempt of the *femAB *deletion mutant to balance osmotic pressure due to the weakened cell wall. In contrast, *opuD *and its homologue, *sa1987*, members of the betaine/carnitine/choline transporter (BCCT) family, were downregulated in BB1305. This latter family of transporters, however, may respond to other kinds of osmotic stress than the *opuC *operon.

Besides quaternary amines or amino acids, solutes such as polyols (e.g. glycerol, arabitol) and sugars (e.g. sucrose, trehalose) may play a role in osmoprotection [44]. Indeed, genes encoding transporters specific for these classes of compounds were also found to be upregulated in BB1305: namely the glycerol uptake facilitator (*glpF*), the sucrose-specific IIBC component of the phosphotransferase system (PTS) (*scrA*), and a hypothetical protein similar to ScrA (*sa0186*). Other genes encoding sugar transporters were also induced in BB1305, i.e. *sa0208/sa0209 *(permease homologue of a maltose/maltodextrin ABC transporter), *sa0260 *(hypothetical protein similar to the ribose transporter RbsU), and *sa0318/sa0320 *(hypothetical protein similar to the pentitol-specific PTS transporter SgaT/SgaA). Considering the observed upregulation of *glpF*, it is noteworthy that *glpD *(aerobic glycerol-3-phosphate dehydrogenase), *glpQ *(glycerophosphoryldiester phosphodiesterase), and *sa0220 *(hypothetical protein similar to GlpQ) also appeared to be upregulated in BB1305. These may support osmoprotection as GlpQ catalyzes the conversion of sn-glycero-3-phosphocholine to glycerol-3-phosphate and choline, the latter of which is oxidized to glycine betaine, which is not metabolized further in osmotically stressed *S. aureus *[45].

#### Stress response and virulence factors

Given the temperature sensitivity of BB1305, a connection to the heat shock regulon was conceivable. In fact, the gene encoding the chaperone DnaK was downregulated according to the microarray data. DnaK belongs to the HrcA and CtsR controlled heat shock regulon in *S. aureus *[46]. A *dnaK*-specific probe, revealed a 3.5 kb-transcript in Northern blot analysis, most probably covering the *hrcA *operon, and confirmed reduced transcription levels in BB1305 at 37°C (data not shown).

**Figure 8:**
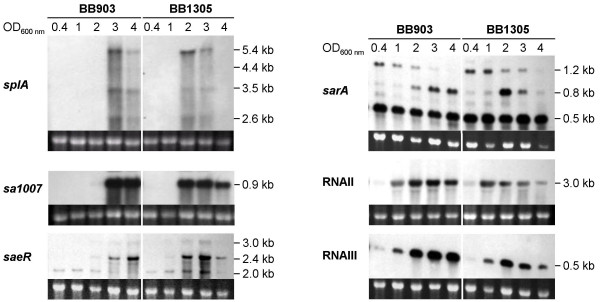
Northern blots of differentially expressed genes in the wild type BB903 and the *femAB*+ backcross BB1305. Cells were harvested at different optical densities as indicated. The amount of RNA loaded onto the respective gels is displayed by the ethidium bromide stained 16S rRNA bands.

Unfavourable environmental conditions are known to induce lysogenic phages, and the upregulation of phage-related genes in BB1305 is most likely to be regarded in the context of a stress response which occurred in the *femAB *deletion mutant AS145. Various stress conditions have also been described to trigger the expression of virulence factors [21]. In BB1305, the transcriptional changes observed included the upregulation of a whole series of virulence genes, such as those coding for lipase (*lip*), urease (*ure*),α-hemolysin precursor (*sa1007*), truncated β-hemolysin (*sa1752 *and *sa1811*), serine proteases (*spl*), cysteine protease (*sspB*) and aureolysin (*aur*). Since the expression of virulence factors depends on a complex regulatory network, this finding prompted us to analyze the transcription profiles of the major global regulators including *sarA *(staphylococcal accessory regulator), *sae *(*S. aureus *exoprotein expression) and the *agr *(accessory gene regulator) operon by Northern blot during growth. In accordance with the expression pattern of two representative virulence factors, namely the α-hemolysin precursor (*sa1007*) and the serine proteases (*splABCDEF*), the regulators and especially RNAIII and *saeRS *peaked at a lower OD_600 _in the *femAB*+ backcross BB1305 than in BB903. At the transition to stationary phase, at an OD_600 _of 4, they had already become clearly weaker in the mutant than in the wild type (Figure 8) revealing a remarkably altered timing in the transcription of the main global regulators in BB1305.

## Conclusion

This is the first thorough characterization of compensatory effects triggered by a shortened pentaglycine interpeptide, the low ability of PBP to crosslink this altered peptidoglycan, and selection for survival. The poorly crosslinked cell wall may be not strong enough to withstand the cells' high internal turgor. This imbalance must have been sensed by the cells, which found a way to counteract osmotic stress, though at the cost of a decreased growth rate and temperature sensitivity. The rearrangements required also involved changes in the expression of metabolic pathways, especially of the arginine-deiminase pathway and the nitrogen metabolism, and seem to be maintained in a stable manner, since they persisted after restoration of the pentaglycine interpeptide. This demonstrates the vast extent of the compensatory adaptations. Such compensatory adaptations following mutagenesis may happen much more frequently than anticipated, and may be the cause of the often observed experimental inability to fully complement mutations with the original wild type alleles.

Cell wall-deficient forms (L-forms) of *S. aureus *are able to survive without an intact murein sacculus, and to internalize and persist in macrophages [47]. Adaptive responses to L-forms of *S. aureus *have recently been described by Fuller et al. [48], who selected cell wall-deficient mutants with subinhibitory concentrations of penicillin in the presence of elevated osmolality. Similar to what we observed, namely that the reconstituted strain BB1305 displayed a higher, more homogeneous oxacillin resistance than the wild type, recovery of the cell wall by the L-forms resulted in a stably inherited penicillin resistance that was independent of a β-lactamase or *mecA*. Apparently, the expected negative effect of reduced *fmtC *or *glnA *expression on oxacillin resistance was compensated in BB1305. A phenomenon, that was also observed with other *fem*, *aux *or *fmt *MRSA mutants which still harboured their original mutations but regained resistance by compensatory events when grown in the presence of β-lactams [18,49].

The temperature sensitivity and the increased susceptibility to bacitracin and teicoplanin are indications for permanent alterations in the membrane. Membrane defects may also be the cause for the premature upregulation of global regulators which in turn most probably triggered the enhanced expression of virulence factors [50,51].

It is likely that the adaptations represent just one of many ways that *S. aureus *could respond to *femAB *inactivation. Some of the findings may be secondary effects caused by slow growth and changes within the cell, not contributing to survival, similar to the upregulation of the purine operon in vancomycin intermediate resistant *S. aureus *[52], that did not contribute to increased glycopeptide resistance [53].

At this stage, we cannot confirm that the numerous adaptive events that occurred in the *femAB *deletion mutant and that are reflected in the *femAB+ cis*-complemented strain were indeed due to mutation(s). The phenotypic adaptations revealed here, such as temperature sensitivity, slowed down growth, but no changes in autolytic activities, were strikingly similar to the phenotype caused by mutations in *femA *after selection for lysostaphin resistant clones [54], which might be evidence for a common survival strategy.

*S. aureus *has shown here a remarkable ability to compensate and survive a severe condition, such as *femAB *inactivation, which prevents peptidoglycan crosslinking. Implementation of potential FemAB inhibitors may therefore entail selection of resistant subpopulations having unwanted characteristics, making a combined treatment with other antibiotics highly advisable.

## Methods

### Bacterial strains and growth conditions

The strains used in this study are listed in Table 1. Bacteria were cultivated either on sheep blood agar or in Luria Bertani (LB) broth (Becton Dickinson, Sparks, MD) at 37°C, unless stated otherwise. The ratio between broth and culture flask volume was 1:5 and incubation was carried out with shaking at 180 rpm.

### Scanning electron microscopy (SEM)

Strains grown in brain heart infusion (BHI) broth at 30°C for 3 h were used to inoculate 1 ml BHI broth in 24-well plates containing polyethylene terephthalate (Thermanox) disks (Life Technologies, Basel, Switzerland) to an OD_600 _of 0.05. The cells were incubated in stationary culture at 30°C for 2 h. Fixation and SEM were carried out as described previously [55].

### Peptidoglycan analysis

Insoluble peptidoglycan was purified from cultures grown to mid-log phase in BHI broth using a standard procedure [56,57]. After removal of teichoic acids by hydrofluoric acid, the relative glycan chain lengths were determined in the peptidoglycan preparations as described elsewhere [58] and their muropeptide patterns were compared following digestion with muramidase [56].

### Antibiotic gradient plates

Qualitative differences in resistance levels were evaluated by swabbing 0.5 McFarland-standard cell suspensions, prepared from freshly grown overnight cultures, along an antibiotic gradient on rectangular LB agar plates. Gradient plates were incubated at 35°C for 24 to 48 h.

### Population analysis profile

Overnight cultures were diluted in 0.85% NaCl and aliquots of 0.1 ml were spread onto LB agar plates containing various oxacillin concentrations. Colony forming units (CFUs) were determined after 48 h.

### Molecular biological methods

General molecular biology techniques were performed as described by Sambrook et al. [59] and Ausubel et al. [60].

### Transcriptional profiling

Overnight cultures were diluted 100-fold in LB broth and cells were grown to an OD_600 _of 0.8–1.0. The cultures were stabilized by incubation with 2 volumes of RNAprotect Bacteria Reagent (QIAGEN, Hilden, Germany) for 5 min at room temperature. Subsequently, cells were harvested by centrifugation, lysed in the presence of 400 μg/ml lysostaphin (Sigma-Aldrich, Taufkirchen, Germany) and total RNA was isolated using the RNeasy Midi Kit (QIAGEN) following the manufacturers' instructions.

Three independent RNA preparations of each strain were reverse transcribed twice, using either cyanine-3' (Cy3) or cyanine-5' (Cy5) as a label. Briefly, 10 μg of total RNA were transcribed into cDNA using Superscript II reverse transcriptase (Invitrogen, Karlsruhe, Germany). The transcription reaction was performed in the presence of 0.1 mM Cy3- or Cy5-labelled dCTP (Perkin Elmer Life Science, Mechelen, Belgium) in addition to 0.2 mM dCTP, 0.5 mM dATP, dGTP and TTP, 75 μg/ml random hexamer primer (Amersham, Bioscience, Freiburg, Germany) and 4U/μl RNase-Out (Invitrogen). RNA was degraded by alkaline hydrolysis at 65°C and cDNA was purified using the MinElute PCR Purification Kit (QIAGEN).

Differentially labelled cDNAs of both strains were competitively hybridized with a custom PCR product microarray (Scienion, Berlin, Germany) resulting in a total of six chips. The microarray contained 2332 open reading frames (ORFs) of the *S.aureus *N315 genome, each represented by adjacent duplicate spots. Hybridization was performed at 42°C for 72 h according to the manufacturer's instructions. The hybridized microarrays were read out with a GenePix 4000B scanner (Axon Instruments/Distribution by Biozyme, Oldendorf, Germany). Image analysis and acquisition of relative data were conducted using GenePixPro 4.1 software (Axon Instruments).

### Microarray data analysis

First, the intensity data arising from the six two-colour spotted microarrays were calibrated and normalized in order to remove systematic technical variation (e.g. different labelling efficiencies and scanning properties of the Cy3 and Cy5 dyes) and to ensure that observed differences in intensities indeed reflect biological signal. Two-channel normalization was performed to adjust the centre and spread of the distribution of intensity log-ratios [61] using the default method of the "marray" package [62] in Bioconductor version 1.8 [63]. Adaptive location normalization within print-tip groups using robust local regression [64] allows the capture of non-linear dependencies of the intensity log-ratio on overall intensity, while ensuring that the computed normalization values are not driven by a small number of differentially expressed genes with extreme log-ratios. Due to scale differences between the arrays we also conducted global scale normalization across arrays.

For identification of differentially expressed genes between the *femAB*+ backcross BB1305 and the wild type BB903, the linear modelling features of the "limma" R package version 2.9.1 were used [65]. In the present experiment, three BB1305 RNA preparations were to be compared with three BB903 RNA preparations using six arrays, i.e. each RNA appeared on two different arrays. Note that technical replicates are not independent: in fact they are likely to be positively correlated. Since the experimental design did not arrange the arrays in groups of biological replicates, we fitted a model with a coefficient for each RNA preparation. The duplicate spots in adjacent position were taken into account by estimating a common value for the intra-duplicate correlation [66] that was used when fitting a linear model for each gene. Finally, we extracted the contrast referring to the average expression differences between the two investigated *S. aureus *strains and computed moderated *t*-statistics using empirical Bayes methods. These borrow information across genes and thus stabilize the analysis even for a small number of arrays [9]. The reported list of potentially interesting genes was determined by adjusting the *p*-values for multiple testing. Here we have chosen the FWER-based *p*-value adjustment according to Holm [10], where the multiple significance level α was set to 0.05.

### Reporter metabolite analysis

The microarray data were further analyzed by a recently developed algorithm that uses the topology of an organism's metabolic network to uncover underlying metabolism-related transcriptional regulation [67]. This algorithm first converts a genome-scale metabolic network of *S. aureus *N315 [11] into a bipartite metabolic graph. In this graph, each metabolite node is then scored based on the normalized transcriptional response of its neighbouring enzymes. Using the genes' *p*-values as inputs to score the enzyme nodes, the algorithm identifies so-called reporter metabolites, designating metabolites around which the most significant transcriptional changes occur.

### Northern blots

The transcription of a selection of genes was verified by Northern hybridization and primers used for probe amplification are listed in Table 4. Overnight cultures were diluted 100-fold in LB broth and incubated for 2 h. The pre-cultures were then diluted in LB broth to an OD_600 _of 0.05 and grown until they reached the desired OD_600_. Total RNA was extracted according to the method of Cheung et al. [68]. For Northern hybridization, 8 μg of total RNA per sample were loaded on a 1.5% agarose gel containing 20 mM guanidine thiocyanate in 1xTris-Borate-EDTA running buffer [69]. Blotting of the electrophoretically separated RNA and detection of transcripts were carried out as described earlier [70].

**Table 4:** Primers used for construction of DIG-labelled DNA probes

**Primer**	**Sequence (5'-3')**
*dltA-*F	TCAGGCGGTACATTAAATCTTGT
*dltA-*R	TATGTGTTGTAAATCGTCGCACT
*dnaK*-F	CGATGAGCCAAAAGTAATTC
*dnaK*-R	TACTTCGAATACACCGTCAC
*fmtC*-F	CCGTATGTCCTTAGTGTTAC
*fmtC*-R	GCAGTACAATCCTACAAAAC
*glnA*-F	AGATGGAACACCATTTGAAG
*glnA*-R	AAACGTTAAAGTGCATACCG
*glpF*-F	TAGACGGAAGTTTTGATTGG
*glpF*-R	GGCAATTGGTCCTAAGATAG
*opuCC*-F	TTGTCGTGTTTGTCTTATCG
*opuCC*-R	ACGTATTCGCAAAACCATAC
*pyrP-F*	TTATCACGGGATTAAGTACG
*pyrP-R*	ACAATCGGAATCATTACAAG
*pyrR-F*	AACGTACAGTGACGAGAATC
*pyrR-R*	TAACTGCATTTCTTTGATCC
RNAII-F	CGAAGACGATCCAAAAC
RNAII-R	TTATCTAAATGGGCAATGAGT
RNAIII-F	GTGATGGAAAATAGTTGATGAG
RNAIII-R	GTGAATTTGTTCACTGTGTCG
*sa1007*-F	TAATGAATCCTGTCGCTAAT
*sa1007*-R	TTCAGTGTATGACCAATCGAA
*saeR*-F	GACCCACTTACTGATCGTG
*saeR*-R	CCTAATCCCCATACAGTTGTG
*sarA*-F	AGGGAGGTTTTAAACATGGC
*sarA*-R	CTCGACTCAATAATGATTCG
*splA*-F	GAATTACCTGGTTGTGCATACG
*splA*-R	GAAGACCTTGCGATAGTTCATG

### Urease assay

McFarland 0.5-standard cell suspensions were diluted 100-fold in urea medium [71] and were incubated in 96-well plates at 37°C for 24 hours.

## Authors' contributions

JH carried out phenotypic characterizations and Northern blots, experimentally validated the microarray data, contributed to the interpretation of the results, and drafted the manuscript. AJ carried out the microarrays. OK performed the reporter metabolite analysis. JS performed the statistical analyses. PAM conducted the peptidoglycan analyses. LGH carried out the scanning electron microscopy. GB participated in the design of the microarray experiments and their interpretation. MH supervised the reporter metabolite analysis and interpreted its results. BBB conceived, designed, and coordinated the study, and participated in writing of the manuscript. All authors read and approved the final manuscript.

## Supplementary Material

Additional file 1 Genes found differentially expressed in the *femAB+ *backcross BB1305 compared to the wild type BB903. The selection of regulated genes is based on statistical significance of moderated *t*-scores and thus also includes genes with a log_2_(fold change) < |1|. The genes are clustered according to their cellular main role as in the *S. aureus *N315 genome annotation on the DOGAN website [73]. Click here for file
