# Supplementary material for: Living with an imperfect cell wall: compensation of femAB inactivation in Staphylococcus aureus
Source: BMC Genomics. 2007 Sep 4;8:307. doi: 10.1186/1471-2164-8-307 (PMC2045680; doi:10.1186/1471-2164-8-307)
Supplement: Additional file 1 — Genes found differentially expressed in the femAB+ backcross BB1305 compared to the wild type BB903. The selection of regulated genes is based on statistical significance of moderated t-scores and thus also includes genes with a log2(fold change) < |1|. The genes are clustered according to their cellular main role as in the S. aureus N315 genome annotation on the DOGAN website [73]. [file 1471-2164-8-307-S1.pdf]

Additional file 1. Genes found differentially expressed in the *femAB+* backcross BB1305 compared to the wild type BB903. The selection of regulated genes is based on statistical significance of moderated *t*-scores and thus also includes genes with a $\log_2$(fold change) < |1|. The genes are clustered according to their cellular main role as in the *S. aureus* N315 genome annotation on the DOGAN website (http://www.bio.nite.go.jp/dogan/MicroTop?GENOME_ID=n315, as of September 24, 2006).

CATEGORY<br>Process<br>ORF ID	Gene	Product	$\log_2$(fold change)	*p*-value
**CELL ENVELOPE AND CELLULAR PROCESSES**				
**Cell wall**				
down				
SA0793	*dltA*	D-alanine-D-alanyl carrier protein ligase	-1.03	0.02
SA1283	*pbpB*	PBP2	-0.47	0.04
**Membrane bioenergetics (electron transport chain and ATP synthase)**				
down				
SA0937		cytochrome D ubiquinol oxidase subunit 1 homologue	-0.70	<0.005
up				
SA0210		hypothetical protein, similar to NADH-dependent dehydrogenase	1.84	<0.005
SA0211		hypothetical protein, similar to NADH-dependent dehydrogenase	1.65	<0.005
**Transport / binding proteins and lipoproteins**				
down				
SA0617	*vraG*	ABC transporter permease	-0.80	0.04
SA0640		hypothetical protein, similar to ABC transporter required for expression of cytochrome bd	-0.57	0.04
SA0655	*fruA*	fructose specific permease	-1.28	0.01
SA0691		lipoprotein, similar to ferrichrome ABC transporter	-0.77	0.02
SA0794	*dltB*	DltB membrane protein	-1.30	0.01
SA0796	*dltD*	poly(glycerophosphate chain) D-alanine transfer protein	-1.38	<0.005
SA0950	*potA*	spermidine/putrescine ABC transporter, ATP-binding protein homologue	-0.43	0.03
SA1042	*pyrP*	uracil permease	-2.37	<0.005
SA1183	*opuD*	glycine betaine transporter	-0.83	0.03
SA1269		Blt-like protein	-1.47	<0.005
SA1270		hypothetical protein, similar to amino acid permease	-1.10	0.01
SA1505	*lysP*	lysine-specific permease	-0.92	0.01
SA1893		lipoprotein precursor	-0.75	0.02
SA1987		glycine betaine transporter *opuD* homologue	-1.07	0.02
SA2303		hypothetical protein, similar to membrane spanning protein	-1.50	0.01
SA2339		hypothetical protein, similar to antibiotic transport-associated protein	-1.68	<0.005
up				
SA0186		hypothetical protein, similar to sucrose phosphotransferase enzyme II	1.40	0.02
SA0208		maltose/maltodextrin transport permease homologue	1.49	<0.005
SA0209		maltose/maltodextrin transport permease homologue	2.02	<0.005
SA0260		hypothetical protein, similar to ribose transporter RbsU	0.66	0.01
SA0318		hypothetical protein, similar to transport protein SgaT	1.53	<0.005

CATEGORY				
**Process**				
**ORF ID**	**Gene**	**Product**	**log$_2$(fold change)**	***p*-value**
SA0320		hypothetical protein, similar to PTS fructose-specific enzyme IIBC component	2.26	<0.005
SA0599		ATP-binding cassette transporter A	0.45	0.04
SA1140	*glpF*	glycerol uptake facilitator	1.39	<0.005
SA2167	*scrA*	PTS system, sucrose-specific IIBC component	0.70	0.02
SA2235	*opuCC*	glycine betaine/carnitine/choline ABC transporter (osmoprotec) *opuCC*	0.90	0.01
SA2426	*arcD*	arginine/ornithine antiporter	1.37	<0.005
**INFORMATION PATHWAYS**				
**DNA modification and repair**				
up				
SA1512		hypothetical protein, similar to formamidopyrimidine-DNA glycosylase	0.51	0.04
**DNA recombination**				
up				
SA1313		probable ATP-dependent DNA helicase RecQ	0.61	0.02
**DNA replication**				
up				
SA1525	*dnaE*	DNA polymerase III, alpha chain	0.66	<0.005
**Protein folding**				
down				
SA1409	*dnaK*	DnaK protein (HSP70)	-0.71	<0.005
up				
SA1659	*prsA*	peptidyl-prolyl cis/trans isomerase homologue	1.03	0.01
**Protein synthesis**				
down				
SA0009	*serS*	seryl-tRNA synthetase	-0.75	0.02
SA0475	*lysS*	lysyl-tRNA synthetase	-0.56	<0.005
SA0496	*rplA*	50S ribosomal protein L1 (BL1)	-0.62	0.04
SA0959		GTP-binding elongation factor homologue	-0.76	0.03
SA1456	*aspS*	aspartyl-tRNA synthetase	-0.86	0.03
SA2041	*rpsC*	30S ribosomal protein S3	-0.73	0.01
**RNA synthesis**				
down				
SA0501	*rpoC*	RNA polymerase beta-prime chain	-0.55	0.01
SA1041	*pyrR*	pyrimidine operon repressor chain A	-1.80	<0.005
SA1956		lytic regulatory protein truncated with Tn*554*	-0.82	0.03
up				
SA0187		hypothetical protein, similar to transcription regulator	1.90	<0.005
SA0321		hypothetical protein, similar to transcription antiterminator BglG family	2.52	<0.005
SA1339	*malR*	maltose operon transcriptional repressor	2.07	<0.005
SA1700	*vraR*	two-component response regulator	0.38	0.05
SA1847	*scrR*	sucrose operon repressor	0.63	0.01
SA2295	*gntR*	gluconate operon transcriptional repressor	1.49	0.02
SA2320		hypothetical protein, similar to regulatory protein PfoR	1.08	0.03
SA2424		hypothetical protein, similar to transcription regulator Crp/Fnr family protein	1.28	<0.005

CATEGORY<br>Process<br>ORF ID	Gene	Product	log_2(fold change)	*p*-value
**INTERMEDIARY METABOLISM**				
**Metabolism of lipids**				
down				
SA0548	*mvaD*	mevalonate diphosphate decarboxylase	-0.62	0.02
SA0843	*fab*	3-oxoacyl-[acyl-carrier-protein] synthase	-0.81	0.03
SA1073	*fabD*	malonyl CoA-acyl carrier protein transacylase	-0.60	0.01
SA1074	*fabG*	3-oxoacyl-(acyl-carrier protein) reductase	-0.65	<0.005
up				
SA0220		hypothetical protein, similar to glycerophosphodiester phosphodiesterase	0.84	<0.005
SA0820	*glpQ*	glycerophosphoryl diester phosphodiesterase	0.59	0.03
SA2349	*crtM*	squalene synthase	0.79	0.01
**Metabolism of amino acids and related molecules**				
down				
SA1150	*glnA*	glutamine-ammonia ligase	-1.03	<0.005
SA1271		threonine deaminase IlvA homologue	-0.99	<0.005
SA1272		alanine dehydrogenase	-1.14	<0.005
SA1608	*metK*	S-adenosylmethionine synthetase	-0.49	0.04
SA1858	*ilvD*	dihydroxy-acid dehydratase	-0.80	0.03
SA1859	*ilvB*	acetolactate synthase large subunit	-1.18	0.05
SA2125		hypothetical protein, similar to formiminoglutamase	-0.79	0.04
up				
SA1012	*argF*	ornithine carbamoyltransferase	1.57	<0.005
SA2082	*ureA*	urease gamma subunit	1.73	<0.005
SA2086	*ureF*	urease accessory protein UreF	0.88	0.01
SA2121	*hutI*	imidazolonepropionase	1.39	0.04
SA2122	*hutU*	urocanate hydratase	2.00	<0.005
SA2318		hypothetical protein, similar to L-serine dehydratase	0.85	<0.005
SA2319		hypothetical protein, similar to beta-subunit of L-serine dehydratase	0.69	0.01
SA2425	*arcC*	carbamate kinase	1.49	<0.005
SA2427	*arcB*	ornithine transcarbamoylase	1.65	<0.005
SA2428	*arcA*	arginine-deiminase	2.04	<0.005
**Metabolism of carbohydrates and related molecules**				
down				
SA1088	*sucC*	succinyl-CoA synthetase (beta subunit)	-0.99	<0.005
SA1089	*sucD*	succinyl-CoA synthetase (alpha subunit)	-0.91	<0.005
up				
SA0143	*adhE*	alcohol-acetaldehyde dehydrogenase	0.85	0.01
SA0258	*rbsK*	probable ribokinase	0.71	<0.005
SA0304	*nanA*	N-acetylneuraminate lyase subunit	1.73	<0.005
SA0305		hypothetical protein, similar to glucokinase	1.50	<0.005
SA0433		alpha-glucosidase	1.53	0.01
SA1142	*glpD*	aerobic glycerol-3-phosphate dehydrogenase	1.10	0.04
SA1338	*malA*	alpha-D-1,4-glucosidase	1.17	<0.005
SA1556	*acuC*	acetoin utilization protein	1.71	0.04
SA2294	*gntK*	gluconokinase	1.66	<0.005
SA2304	*fbp*	fructose-bisphosphatase	0.90	0.01

CATEGORY<br>Process<br>ORF ID	Gene	Product	$\log_2$(fold change)	*p*-value
**Metabolism of coenzymes and prosthetic groups**				
up				
SA1259	*dfrA*	dihydrofolate reductase	0.62	<0.005
**Metabolism of nucleotides and nucleic acids**				
down				
SA0016	*purA*	adenylosuccinate synthase	-1.13	0.03
SA1043	*pyrB*	aspartate transcarbamoylase chain A	-2.02	<0.005
SA1044	*pyrC*	dihydroorotase	-2.67	<0.005
SA1046	*pyrAB*	carbamoyl-phosphate synthase large chain	-1.85	0.01
SA1048	*pyrE*	orotate phosphoribosyltransferase	-1.78	0.01
up				
SA0131	*pnp*	purine nucleoside phosphorylase	0.83	<0.005
SA1013		hypothetical protein, similar to carbamate kinase	2.18	0.01
SA2410	*nrdD*	anaerobic ribonucleoside-triphosphate reductase	0.96	<0.005
**NO SIMILARITY**				
up				
SA0663		hypothetical protein	1.13	<0.005
SA1210		hypothetical protein	0.58	0.02
SA1476		hypothetical protein	1.95	<0.005
**OTHER FUNCTIONS**				
**Adaption to atypical conditions**				
up				
SA0147	*capD*	capsular polysaccharide synthesis enzyme Cap5D	0.81	<0.005
**Detoxification**				
up				
SA1238		hypothetical protein, similar to tellurite resistance protein	0.54	0.04
**Miscellaneous**				
down				
SA1193	*fmtC*	oxacillin resistance-related FmtC protein	-1.12	<0.005
**Pathogenic factors (toxins and colonization factors)**				
down				
SA0587		lipoprotein, streptococcal adhesin PsaA homologue	-0.55	0.01
up				
SA0091	*plc*	1-phosphatidylinositol phosphodiesterase precursor	1.69	0.02
SA0900	*sspB*	cysteine protease precursor	1.07	0.02
SA1007		alpha-hemolysin precursor	1.64	0.02
SA1630	*splB*	serine protease SplB [Pathogenicity island SaPIn3]	2.48	<0.005
SA1752	*(hlb)*	truncated beta-hemolysin	1.96	0.01
SA1811	*(hlb)*	truncated beta-hemolysin	2.06	<0.005
SA1813		hypothetical protein, similar to leukocidin chain lukM precursor	1.64	0.04
SA2430	*aur*	zinc metalloproteinase aureolysin	2.05	0.01
SA2463	*lip*	triacylglycerol lipase precursor	1.68	<0.005

CATEGORY<br>Process<br>ORF ID	Gene	Product	log_2(fold change)	*p*-value
**Phage-related functions**				
up				
SA0252	*lrgA*	holin-like protein LrgA	1.24	0.03
SA0754		hypothetical protein, similar to lactococcal prophage ps3 protein 05	1.26	0.01
SA1782		hypothetical protein [Bacteriophage phiN315]	1.92	<0.005
SA1785		hypothetical protein [Bacteriophage phiN315]	2.27	<0.005
SA1786		hypothetical protein [Bacteriophage phiN315]	2.29	<0.005
SA1797		hypothetical protein [Bacteriophage phiN315]	2.56	<0.005
SAS063		hypothetical protein [Bacteriophage phiN315]	2.08	<0.005
**Transposon and IS**				
up				
SA2387	*tnpB*	transposition regulatory protein TnpB - *S. aureus* transposon Tn*554*	1.21	0.04
**SIMILAR TO UNKNOWN PROTEINS**				
down				
SA0100		conserved hypothetical protein	-0.66	<0.005
SA0477		conserved hypothetical protein	-1.47	<0.005
SA0701		conserved hypothetical protein	-0.51	0.03
SA1536		conserved hypothetical protein	-0.72	0.01
SA2143		conserved hypothetical protein	-0.82	0.02
SA2305		conserved hypothetical protein	-0.67	0.03
SA2328		conserved hypothetical protein	-0.42	0.02
up				
SA0184		conserved hypothetical protein	2.35	<0.005
SA0185		conserved hypothetical protein	2.43	<0.005
SA0789		conserved hypothetical protein	0.54	0.04
SA1014		conserved hypothetical protein	1.54	<0.005
SA1258		conserved hypothetical protein	0.43	0.04
SA1661		conserved hypothetical protein	0.45	0.05
SA1690		conserved hypothetical protein	0.60	0.01
